# Aripiprazole for treatment of attenuated psychosis syndrome in an adolescent with 22q11.21 deletion syndrome: A case report

**DOI:** 10.1097/MD.0000000000046652

**Published:** 2025-12-12

**Authors:** Norah K. Algarzae, Samah H. Alkhawashki, Aqeel T. Alkhiri, Maha Algazlan, Mohammed A. Alhassan, Waled Albalawi, Wedad S. Sarawi

**Affiliations:** a Department of Physiology, College of Medicine, King Saud University, Riyadh, Saudi Arabia; b Department of Psychiatry, College of Medicine, King Saud University, Riyadh, Saudi Arabia; c Department of Psychiatry, King Saud University Medical City, King Saud University, Riyadh, Saudi Arabia; d Clinical Sciences Department, College of Medicine, Princess Nourah bint Abdulrahman University, Riyadh, Saudi Arabia; e Department of Medical Specialties, College of Medicine, Majmaah University, Al Majmaah, Saudi Arabia; f Department of Pharmacology and Toxicology, College of Pharmacy, King Saud University, Riyadh, Saudi Arabia.

**Keywords:** 22q11.2 deletion syndrome (22q11DS), adolescent, aripiprazole, attenuated psychosis syndrome (APS)

## Abstract

**Rationale:**

The 22q11.2 deletion syndrome is a prevalent genetic condition associated with an increased risk of psychotic disorders. This case report describes an adolescent girl diagnosed with 22q11.2 deletion syndrome at the age of 4 years. She had a history of developmental delays, hearing impairments, and orthopedic complications requiring multiple surgeries.

**Patient concerns:**

At 15 years of age, she had a 2-month history of abnormal beliefs, perceptual disturbances, behavioral changes, and irritability.

**Diagnoses:**

She was diagnosed with attenuated psychosis syndrome.

**Interventions:**

She was treated with aripiprazole as a therapeutic intervention coupled with cognitive behavioral therapy, leading to a significant improvement in her symptoms; however, she developed akathisia and was therefore switched to risperidone, which was better tolerated in the long run.

**Outcomes:**

This case emphasizes the need for early intervention and personalized, tailored treatment options that can address the unique needs and vulnerabilities of these patients with complex genetic conditions and associated psychiatric manifestations.

**Lessons:**

This case report represents the therapeutic success achieved with aripiprazole, demonstrating the potential effectiveness of this medication and its potential to improve patient outcomes when it is coupled with cognitive behavioral therapy, highlights the vulnerability of the patient group to antipsychotic-related side effects, and underscores the need for further development of optimized treatment strategies.

## 1. Introduction

A microdeletion through the de novo deletion route causes the genetic disease known as the 22q11.2 deletion syndrome (22q11DS).^[1]^ In 22q11DS a patient’s prognosis is affected by several physiological changes including gastrointestinal, renal, cognitive, and mental abnormalities.^[2]^ According to an international brain and behavior consortium, 22q11DS is considered the most prominent known molecular risk factor for schizophrenia.^[3]^ The reported incidence occurs in 1/2000 to 1/4000 live births.^[3,4]^ 22q11DS is also associated with neuropsychiatric presentations, including attention deficit hyperactivity disorder, found in 37.10% of children and adolescents with 22q11DS.^[3]^ Although there was a significant male patient preponderance, female patients were more likely to suffer from anxiety and unipolar mood disorders.^[3]^ Risk factors for neuropsychiatric diseases with variable clinical and cognitive presentations, including attention deficit, anxiety disorders, autism spectrum, and psychotic-like traits, are impacted by variations of this gene deletion syndrome.^[5]^

There is a paucity of research on patients with 22q11DS and on their responsiveness to antipsychotic medications.^[2]^ Less research has been done on the substantial correlation between 22q11DS and attenuated psychosis syndrome (APS), a disorder marked by symptoms that are similar to those of psychosis but fall below the threshold.^[2]^ According to a study, the “age effect” of APS in children and adolescents occurs at an optimal cutoff age of 14.7 years for psychosocial functioning and 14 years for positive symptoms; older adolescents, on the other hand, exhibited better functioning and less severe positive symptoms.^[6]^ It has been reported that aripiprazole (15 mg/d) is administered to patients with, 22q11DS alongside other medications including Risperidone (4 mg/d) and Clordemetil-diazepam (2 mg/d).^[7]^

One of the most often administered atypical antipsychotics is aripiprazole, which is effective for several conditions in children and adolescents.^[8]^ Low-dose aripiprazole has been successfully used to treat APS in a patient with 22q11DS.^[9]^ However, the use of aripiprazole necessitates clinical monitoring due to the possibility of adverse effects.^[8]^ A series of 3 cases showed the positive effects of using aripiprazole on 3 different patients with 22q11DS^[10]^; also lurasidone and olanzapine were both safe and effective.^[11–13]^ This case report advances knowledge of possible treatment approaches for APS in the setting of 22q11DS by demonstrating the effective use of aripiprazole as a therapeutic intervention.^[14]^

## 2. Case presentation

### 2.1. Patient description

This is a case of a 15-year-old Saudi girl referred from the endocrinology clinic to the child and adolescent psychiatry clinic at King Saud University Medical City due to aggressive behavior and a suicide attempt. She lives with her parents and 3 siblings in a city near Riyadh, Saudi Arabia. She was born via normal spontaneous vaginal delivery to a woman in a consanguineous marriage, with no known psychiatric or neurological conditions in the family. In terms of her academic and social life, she attends school regularly as an intermediate school student, achieving good performance and socialization.

### 2.2. Case history

In 2021, she informed her mother that someone was trying to cut and touch her hair, and that someone was following her; these hallucinations affected her sleep, and she could not sit calmly with her family. Symptoms worsened, and she became irritable, aggressive, and had suicidal thoughts. This lasted for 2 months, after which her parents took her to a private psychiatrist. She was started on olanzapine, which was subsequently changed to lurasidone because of sedation. Her symptoms improved immensely with medication, and she was able to attend school the year after. However, at school, she had anxiety episodes accompanied by frequent stomachaches, loss of interest, low mood, sleep disturbance, and loss of appetite. During her time at school, she avoided peer interactions, struggled to make friends, avoided public speaking in class, felt constantly embarrassed or humiliated, and was afraid of being judged negatively. She was referred to the child psychiatric clinic for further evaluation and management.

During a psychological review of her symptoms, she was reported to have sensitivity to lights and loud sounds. There was no history of autistic symptoms, attention deficit, hyperactivity, or tics. There were no challenges with mood, including depressive symptoms, mania, or hypomania. At the time of the initial visit, she had been on lurasidone 18.5 mg orally twice daily for 3 months. Her past medical history revealed she was diagnosed with DiGeorge syndrome at age 4, had genu varum with multiple orthopedic correction surgeries and short stature, and was not able to walk in her first few years. She also had precocious puberty and was on lupron until 2 years before this presenting problem. She has hearing impairment with an adverse history of head trauma, seizures, and cardiac conditions.

### 2.3. Assessment

The initial assessment was conducted in the child and adolescent psychiatry clinic at King Saud University Medical City; the patient fulfilled the criteria for other specified schizophrenia spectrum and other psychotic disorders including APS. She was also diagnosed with comorbid social anxiety disorder. Shortly after that, a whole-exome sequencing was repeated and confirmed a de novo pathogenic 22q11.21 spanning the critical 22q11DSregion (17370127–19084594). The Wechsler Intelligence Scale showed verbal = 47, performance = 75, and full scale = 58. The brain magnetic resonance imaging was unremarkable.

### 2.4. Treatment and outcome

The patient showed initial improvement with lurasidone; however, because of the need to consume 300 kcal of food and the unavailability of the medication in the hospital, which has been causing financial strain for the family, she was cross-tapered to aripiprazole 5 mg for 2 weeks, while decreasing lurasidone to 18.5 mg daily, then increased by 2.5 mg increments every week until a dose of 10 mg was reached. Follow-up visits showed stable vitals within the normal range; she weighed 44.8 kg and had a body mass index of 21.31 kg/m^2^. School and socialization performance were relatively good, although there were still episodes of touching her hair, stomachaches, tremors, appearing fidgety, irritability, disturbed sleep, inattention, and easy distractibility. She was referred for cognitive behavioral therapy (CBT) for social anxiety disorder, during which a full workup assessment was completed to fully understand her cognitive and educational functioning skills.

Aripiprazole was continued for 2 years. She was started on escitalopram 5 mg for 5 days at age 17 and then increased it to 10 mg po daily. In her continued appointments, the patient presented later with restlessness, heightened anxiety, and sleep disturbances consistent with akathisia. The management options were discussed with the patient and her family, including the following: adding a beta-blocker (e.g., propranolol), reducing the dose, or switching to another antipsychotic with a lower risk of akathisia. Upon discussion with the family, she was switched to risperidone, which was well-tolerated and resulted in a positive overall response to the symptoms experienced.

## 3. Discussion

This case report illustrates the intricate interaction of environmental, developmental, and genetic factors that resulted in a distinct manifestation of symptoms associated with APS and 22q11DS. This patient’s complicated symptoms and unusual medical history made her management a challenging task requiring a customized therapy strategy considering her psychological, developmental, and medical background. The patient’s initial treatment with olanzapine was poorly tolerated, leading to an unacceptable degree of sedation. She was switched her to lurasidone leading to an initial improvement but due to the high cost of the drug and the need to consume a 300-kcal meal the course of treatment had to be changed. In the new course of treatment, the olanzapine was changed to lurasidone and lurasidone to aripiprazole, indicating the difficulties in practical pharmacological management due to side effects and limitations in accessibility of the drugs.^[14]^

On the new treatment regimen, this patient reported notable improvements in her symptoms after switching to aripiprazole, including a reduction in anxiety, sleep difficulties, and the cessation of aberrant beliefs. Similarly, studies have shown aripiprazole as a frequently well-tolerated and beneficial treatment for psychotic disorders.^[15]^ The patient’s favorable reaction to aripiprazole further demonstrated the significance of regularly evaluating and altering medication by tolerance and responses from patients, which significantly impact compliance and treatment outcome.^[16,17]^ In this case, her active participation in the process contributed to finding a medication regimen that not only helped manage her symptoms but was also feasible for her daily routine.

Despite these improvements, the patient still faced social and academic challenges, thus, the team focused on providing her with an all-encompassing and multifaceted care. Addressing these issues necessitated a thorough evaluation of her cognitive and academic capacities as well as the implementation of CBT for her social anxiety problem. CBT has become a popular and successful psychotherapy in many countries.^[18]^ The complete extent of the 22q11DS’s impact on mental health is yet unknown, although it has been linked to several physical and mental symptoms. Future studies in this field may yield important information for more effectively managing such complicated cases. The road map of this patient highlights the importance of a patient-centered, adaptable, and all-encompassing approach to care, especially for individuals with complicated medical issues. Early identification and intervention of APS in people with 22q11DS is essential. Aripiprazole’s efficacy as a pharmacological intervention for APS in this patient highlights the medication’s potential as a therapy option with regular follow-ups and possible treatment plan modifications in response to patient input and tolerance.

This case report highlights the sensitivity of patients with 22q11DS to antipsychotic-related side effects. Similarly, a systematic review by de Boer et al showed that people with 22q11DS exhibit heightened vulnerability to adverse effects when treated with antipsychotic medications.^[19]^ Furthermore, other studies reported that side effects of akathisia and Parkinsonian symptoms were the most prevalent among this population.^[12]^ These reports confirm the critical importance of close monitoring for adverse effects when prescribing antipsychotics to individuals with 22q11DS. A thorough and multifaceted approach to care beyond drug control is crucial. Further research and clinical follow-up are necessary to understand this constellation of symptoms better and to optimize patient management.

## 4. Conclusion

This case report explores the use of aripiprazole in treating APS in a patient with 22q11DS and coupled it with CBT, shedding light on a lesser-known association and addressing the scarcity of evidence in managing APS in pediatric patients with genetic syndromes. This case report represents the therapeutic success achieved with aripiprazole demonstrating the potential effectiveness of this medication and its potential to improve patient outcomes when its coupled with CBT. Through her unique medical background, this patient highlights the vulnerability of the patient group to antipsychotic-related side effects. In a similar case report, a patient with 22q11DS and schizophreniform disorder was treated with aripiprazole and showed initial positive results, providing a useful reference for the therapeutic rationale we applied and validating the potential efficacy and tolerability of aripiprazole in this genetically vulnerable population.^[20]^ However, in our case, we also incorporated CBT, which further contributes to the broader understanding of available treatment options for APS in the context of 22q11DS. These findings highlight the importance of tailoring interventions to the individual patient and underscore the need for further development of optimized treatment strategies.

## Acknowledgments

The authors are grateful to the team of physicians, nurses and allied health professionals, working at King Saud University Medical Complex who collaborated on this patient’s case.

## Author contributions

**Conceptualization:** Norah K. Algarzae, Samah H. Alkhawashki, Aqeel T. Alkhiri, Maha Algazlan, Mohammed A. Alhassan, Waled Albalawi.

**Data curation:** Norah K. Algarzae, Samah H. Alkhawashki, Aqeel T. Alkhiri, Maha Algazlan, Mohammed A. Alhassan, Waled Albalawi.

**Formal analysis:** Norah K. Algarzae, Samah H. Alkhawashki, Aqeel T. Alkhiri, Wedad S. Sarawi.

**Investigation:** Samah H. Alkhawashki.

**Methodology:** Norah K. Algarzae, Samah H. Alkhawashki, Aqeel T. Alkhiri, Wedad S. Sarawi.

**Project administration:** Aqeel T. Alkhiri.

**Validation:** Wedad S. Sarawi.

**Writing – original draft:** Samah H. Alkhawashki, Aqeel T. Alkhiri, Maha Algazlan, Mohammed A. Alhassan, Waled Albalawi.

**Writing – review & editing:** Norah K. Algarzae, Samah H. Alkhawashki, Aqeel T. Alkhiri, Wedad S. Sarawi.
